# National Policies to Limit Nutrients, Ingredients, or Categories of Concern in School Meals: A Global Scoping Review

**DOI:** 10.1016/j.cdnut.2024.104456

**Published:** 2024-09-19

**Authors:** Emily A Busey, Grace Chamberlin, Kayla Mardin, Michelle Perry, Lindsey Smith Taillie, Francesca R Dillman Carpentier, Barry M Popkin

**Affiliations:** 1 Global Food Research Program, Carolina Population Center, University of North Carolina at Chapel Hill, Chapel Hill, NC, United States; 2 Department of Health Policy and Management, Gillings School of Global Public Health, University of North Carolina at Chapel Hill, Chapel Hill, NC, United States; 3 Department of Nutrition, Gillings School of Global Public Health, University of North Carolina at Chapel Hill, Chapel Hill, NC, United States; 4 Hussman School of Journalism and Media, University of North Carolina at Chapel Hill, Chapel Hill, NC, United States

**Keywords:** school food environment, food policy, school meals, ultra-processed foods, childhood obesity, scoping review

## Abstract

The school food environment is a key intervention point for influencing children’s and adolescents’ diets. As more countries establish school meal programs to provide critical nourishment to students, establishing standards for the foods served can increase the consumption of key nutrients and limit the consumption of foods that do not build health. This global scoping review explores the prevalence and basic characteristics of national policies that regulate food served through school meals across 193 countries, particularly by restricting the provision of categories, nutrients, or ingredients of nutritional concern. We gathered evidence from policy databases, grey literature, peer-reviewed literature, and primary policy documents. We included nationally mandated policies that included restrictions on categories, nutrients, or ingredients of concern served in school meals. Policies that were sub-national, voluntary, and/or did not include restrictive language were excluded from this review. Data was collected in research electronic data capture then extracted into Microsoft Excel and analyzed for policy frequency, prevalence by world region or country income group, and prevalence of certain policy characteristics. Globally, only 15% of countries were found to have a national-level policy restricting foods served through school meals in some capacity, including either nutritional or categorical restrictions. The majority of these policies were found in high-income countries, and no low-income countries had a policy meeting inclusion criteria. Policies in Latin-American and Caribbean countries limited the content of more nutrients of concern than in other regions. Although many policies included explicit guidelines to monitor implementation, few outlined mechanisms for policy enforcement. Future research should evaluate the impact of various school meal regulatory approaches, including implementation of similar policies at sub-national levels, and other elements that affect the impact of school meal programs, such as procurement, infrastructure, costs to school and to students and their families, and acceptability and consumption of foods provided.

## Introduction

Child and adolescent consumption of calorie-dense, ultra-processed foods high in sugar, sodium, and saturated fat is increasing rapidly across low-middle-income countries (LMICs) and remains elevated in higher-income countries (HICs) [1]. Dietary patterns dominated by these foods are linked to a greater risk of overweight and obesity, all-cause mortality, and many other noncommunicable diseases [2,3]. From 1990 to 2022, obesity among children and adolescents increased in virtually every country in the world, with the global prevalence of obesity and overweight now exceeding 390 million children ages 5–19 y and 37 million for children under 5 y [4,5]. Meanwhile, 148 million children under age 5 experienced stunting in 2022, and another 45 million had wasting or severe wasting [6].

As children consume a substantial portion of their daily food intake during school hours, the school food environment and school meals, in particular, offer a prime opportunity for improving and protecting children’s dietary quality and preventing malnutrition from over- or underconsumption [7,8]. Over 400 million children around the world are estimated to receive regular school meals, and this number is expected to grow as more countries implement national school meal programs to improve enrollment and nutrition status [9]. The provision of minimally processed, nutritious foods through school lunch and breakfast programs can contribute to improved health and support the formation of lifelong healthy eating habits.

Establishing nutrition standards for school meals can increase alignment with dietary guidelines for healthy development by promoting adequate consumption of key nutrients and limiting exposure to and consumption of unhealthy foods [10,11]. A meta-analysis of 91 studies examining the impact of different school food environment policies around the world found that setting standards for healthier school meals increases fruit intake, whereas reducing fat and sodium consumption across children’s entire diet, not just foods consumed at schools [12]. In Brazil, participation in the country’s national school meal program [Programa Nacional de Alimentação Escolar (PNAE)] has been associated with lower prevalence of overweight and obesity, increased intake of healthy foods, and overall improvement in diet quality [13,14]. In the United States, strengthened federal nutrition standards for the United States national school lunch program led to increased fruit intake and reduced total fat, saturated fat, and sodium intake and were associated with significant decreases in BMI z-score (BMI in kg/m^2^ adjusted for age and sex) among school-aged children (ages 5–18 years) [12,15]. United States evidence also indicates that strengthening the nutritional requirements for school meals increased dietary quality across socioeconomic groups, suggesting that such interventions can encourage greater equity in nutrition-related health outcomes [16].

In alignment with the WHO guidance to develop regulatory interventions to influence the school food environment, more countries are taking action to regulate the provision of school meal programs [9,17]. Despite global interruptions to school meal provision during the COVID-19 pandemic, the reach of school feeding now exceeds prepandemic levels as national leaders have increasingly recognized the importance of school meals: in 2022, 418 million children received meals at school, >30 million before the pandemic [9]. In the same timeframe, global investment in school meals increased by 5 billion United States dollars, with low-income countries (LIC), in particular, increasing their domestic funding by 15% [9]. Despite this evidence of increasing school meal availability worldwide, there is little documentation of global patterns in regulation, implementation, or impact, particularly regarding national-level mandatory policies that limit the provision of foods that can harm health [12,[18], [19], [20], [21], [22], [23], [24], [25]].

In this scoping review, we aim to document the global prevalence and basic characteristics of national policies to improve the dietary intake of children via school meal programs. Specifically, we aim to *1*) identify countries with any national, mandatory policy to limit the availability of foods or beverages, nutrients, or ingredients of health concern served in a national school meal program and *2*) examine patterns in policy adoption by country income level and world region.

## Methods

We conducted a scoping review to identify and describe national-level, mandatory school food environment policies regulating the provision of unhealthy food or nutrients of concern in school meals in all countries in the world. We registered the protocol for this review in the open science framework on 1 February, 2023 [26]. We followed the PRISMA extension for scoping review guidelines and used previously described search strategies for applying systematic search methods to grey literature [27,28].

### Sample

We included all United Nations member countries as of 12 September, 2022 (*n* = 193) in this review (Supplemental Table 1) [29]. We used the World Bank’s fiscal year 2022–2023 income and region classifications to analyze trends by income level and world region [30].

### Policy inclusion/exclusion criteria

We sought to identify national-level policies that place mandatory nutritional restrictions on food and beverages served in school meals. Policies implemented by sub-national jurisdictions (e.g., states, territories, provinces, or cities) or not containing restrictive language (e.g., focused only on the promotion of healthier food categories) were excluded from this review. (See inclusion and exclusion criteria outlined in Supplemental Table 2).

We defined school meal programs as any government-sponsored program that provides meals to students in public schools on a national scale. We defined “restriction” as a limit or ban on specific foods, food categories, nutrients, or ingredients of concern. Policy language must have indicated clear restrictions (e.g., “prohibited” or “may not be served”) for inclusion. (See these and other key definitions in Table 1). As outlined in the registered review protocol [26], we also gathered data on policies setting certain nutritional standards for competitive foods sold in schools and marketing in schools; these findings are reported in another review [31].

**TABLE 1:** Key terms.

Term	Definition
Policy	Any legislation, regulation, standard, or guideline that meets the inclusion criteria laid out in this search
National policy	Policies adopted by a national government that apply to the entire country
Mandatory policy	Policies that are statutory or mandated by law. This excludes nonbinding recommendations and suggested guidelines (e.g., national dietary guidelines are not included unless a national policy requires a school meal program to adhere to them)
Categorical restriction	Prohibited inclusion/service of a list of specified food or beverage types or categories in the school meal program (e.g., chips, sweets, sweetened carbonated beverages), regardless of each individual food or beverage item’s nutritional profile
Nutrient restriction	Prohibited inclusion/service of foods/beverages based on their individual nutritional content/profile (e.g., grams of sodium or total sugar per specified unit)
Ingredient restriction	Prohibited inclusion/service of foods/beverages containing a specific ingredient. Prohibited ingredients in this review include non-nutritive sweeteners and caffeine
School meals	A meal that is prepared and provided to students at school as part of a national program, either for free or for purchase. This includes breakfast or lunch and excludes foods and drinks available for purchase outside of a formal meal program

We standardized grade levels and ages into the following: *1*) preschools: prekindergarten, children under 6 y of age; *2*) primary schools: grades kindergarten–5th grade or children between 6–11 y of age; *3*) lower-secondary/middle schools: 6th–8th grade or students 11–13 y of age; *4*) upper-secondary/high schools: 9th–12th grade or students 13–18 y of age. We excluded policies that solely targeted “social care” or “childcare” institutions.

### Search and data sources

To identify active policies and primary regulatory documents, we conducted an iterative search process using the following sources: *1*) existing global policy databases (e.g., the World Cancer Research Fund’s NOURISHING database, The WHO’s global database on the implementation of nutrition action [32], and the global obesity observatory); *2*) peer-reviewed literature; *3*) official government websites; *4*) Internet search engines; and *5*) in-country contacts for clarifying ambiguity in policy interpretation or identification. These contacts included staff from national ministries related to food, health, or education, UNICEF, the World Food Program, and faculty from research universities. We found in-country contacts via government websites, authors of relevant scholarly articles, or personal academic connections. We used peer-reviewed articles and grey literature to identify and validate the existence or absence of policies and their evolution over time if policies were not immediately located through a search.

For each country, we identified any policies possibly meeting inclusion criteria in existing global policy databases. If we found a policy of interest for a country, we next sought primary policy documentation either via the database, using an internet search engine, or other sources listed above. If a country had no policy listed in global databases, we then searched grey and peer-reviewed literature to determine policy existence and, where applicable, locate primary policy documentation. These steps were repeated for each country (Supplemental Figure 1).

When using search engines to identify policies, search terms for each country included (school or cafeteria or canteen) AND (food or beverage or nutrition) AND (policy or regulation or restriction or guidelines) AND (school meals or school food). (See example search described in Supplemental Text 1). These searches were repeated for each country between 1 August, 2022 and 30 June, 2023.

We placed no limits on the language or publication date of the source in order to capture as much information as possible, utilizing the following methods to translate and interpret policies written in languages other than English: *1*) Google Translate: We used the Google Translate text, website, and document functions to translate documents; *2*) Translators: We consulted native speakers to translate and interpret documents when available; *3*) Corroborated information: We compared translated policies to information found in other review sources (e.g., from policy databases). If doubt remained about the accuracy of translation or interpretation of policy wording/meaning after completion of these 3 steps, we reached out to researchers and/or ministry officials in the country of interest for clarification. After these steps, we discussed any unresolved questions as a team and, after reaching a consensus, coded the policy using all available information. Countries requiring additional clarification from in-country experts are noted in Supplemental Table 1.

### Data collection

We developed a codebook in collaboration with researchers with expertise in marketing regulations and school food policies (Supplemental Table 3). The research team reviewed and piloted the codebook and modified it to meet research objectives and ensure researchers’ responses were consistent across questions. Data extracted from policies includes the existence of school meal restrictions, target grade/age, specific food category, nutritional/ingredient criteria, and the presence of monitoring and enforcement language. Changes to policies after the search timeframe (1 August, 2022–30 June, 2023) were not captured in this dataset.

We used research electronic data capture (REDCap) for data collection [33,34], including REDCap’s double data entry module to assess inter-rater reliability for a random sample of 20 countries. Any discrepancies were reviewed as a group and coded based on consensus. Two coders had 85% agreement across all codebook variables, indicating sufficient inter-rater reliability. They then independently coded the remaining 173 countries and raised any questions for further review with the research team. Upon completion of data collection and coding, we extracted all data from REDCap and analyzed them using Microsoft Excel [35] for global policy prevalence, presence of certain policy characteristics, and policy prevalence by world region or country income group. We represented these findings in figures created using Microsoft Excel and Adobe Illustrator [36].

## Results

### Global overview

Worldwide, 15% of countries (*n* = 29) were found to have a national-level, mandatory policy restricting food categories, nutrients, or ingredients of health concern in a school meal program (Table 2) [[37], [38], [39], [40], [41], [42], [43], [44], [45], [46], [47], [48], [49], [50], [51], [52], [53], [54], [55], [56], [57], [58], [59], [60], [61], [62], [63], [64], [65], [66], [67], [68], [69], [70], [71], [72], [73], [74], [75]]. The remaining countries (*n* = 164) were excluded for not having a policy or for having a policy that was implemented at the sub-national level, was voluntary, or did not restrict the provision of unhealthy foods (see Supplemental Table 2 for examples). The included restrictions specifically limited which foods could be served or provided to students. This does not include foods that students bring from home or foods available for purchase outside of a formal school meal program (e.g., from vending machines or tuck shops). Five of these countries had school meal regulations but no restrictive policies regulating competitive food or marketing in schools, as laid out in the companion article referenced above [31].

**TABLE 2:** Countries with mandatory policies restricting categories, nutrients, or ingredients of health concern served in a national school meal program.

Country	Categorical and/or nutritional criteria for restriction																							
	Specific food or beverage categories	Foods: Nutrients and ingredients profiled/content restricted								Beverages: Nutrients and ingredients profiled/content restricted									School levels covered				Includes monitoring/enforcement	
		Calories	Sugar: total	Sugar: added	Fat: total	Fat: saturated	Fat: *trans*	Sodium	NNS	Calories	Sugar: total	Sugar: added	Fat: total	Fat: saturated	Fat: *trans*	Sodium	Caffeine	NNS	Preschool	Primary	Lower secondary	Upper secondary	Monitoring	Enforcement
Armenia [37]		✓			✓					✓			✓							✓	✓	✓	✓	
Bahamas [38]	✓																		✓	✓	✓	✓	✓	
Barbados [39]	✓	✓		✓	✓	✓	✓	✓										✓	✓	✓	✓	✓	✓	
Belarus [40,41]	✓																		✓	✓	✓	✓	✓	
Brazil [42,43]	✓					✓		✓											✓	✓	✓	✓	✓	
Bulgaria [44,45]	✓			∗	∗						✓	✓	✓			✓	∗	∗	✓	✓	✓	✓		
Cabo Verde [46,47]		∗		✓	✓	✓	✓	✓											✓	✓	✓	✓	✓	✓
Costa Rica [48,49]	✓	∗	∗		∗	∗	∗	∗				✓							✓	✓	✓		✓	✓
Dominican Republic [50]	✓																		✓	✓	✓	✓	✓	
El Salvador [51]	✓			✓	✓	✓	✓	✓	∗			✓	✓	✓	✓	✓		∗	✓	✓	✓	✓	✓	✓
Estonia [52]	✓																		✓	✓	✓	✓		
France [53]			∗	∗	∗														✓	✓	✓		✓	
Hungary [54,55]	✓			✓	∗			✓	✓			✓					∗	✓	✓	✓	✓		✓	
India [56,57]	✓																		✓	✓	✓	✓	✓	
Israel [58,59]	✓		✓			✓		✓			✓			✓		✓				✓				✓
Latvia [60]	✓	✓		✓∗	✓∗			✓∗	✓			✓	✓			✓	✓	✓	✓	✓	✓	✓		
Lithuania [61]	✓				✓			∗	✓								∗		✓	✓	✓	✓	✓	
Malta [62]	✓		∗	∗	∗	∗	∗	∗			∗	∗	∗	∗						✓	✓	✓	✓	✓
Mexico [63]	✓		∗	✓∗	✓∗	✓∗	∗	✓∗				∗						✓		✓	✓		✓	✓
Peru [64]	✓	✓	✓			✓	✓	✓			✓			✓	✓	✓			✓	✓	✓	✓	✓	
Poland [65]	✓				∗			∗			✓								✓	✓	✓	✓	✓	
Portugal [66]	✓																✓		✓	✓	✓	✓		
Qatar [67]	✓	✓	✓	∗	✓	✓	✓	✓		✓	✓	✓	✓	✓	✓	✓	✓	∗		✓	✓	✓	✓	✓
Seychelles [68]				✓	✓	✓		✓				✓	✓	✓		✓			✓	✓	✓	✓	✓	
Slovakia [69]	✓	✓		∗	✓∗			∗	∗	✓		∗					∗	∗	✓	✓	✓	✓		
Slovenia [70,71]		✓	✓		✓	✓	✓	✓			✓		✓	✓	✓	✓			✓	✓	✓	✓	✓	✓
Ukraine [72]	✓	✓	✓∗	✓∗	✓∗	✓∗	✓	∗	✓		✓	✓				✓	✓	✓	✓	✓	✓	✓	✓	
United States [73]		✓				✓	✓	✓					✓							✓	✓	✓	✓	✓
Uruguay [74,75]		✓	✓		✓	✓	✓	✓		✓	✓		✓	✓	✓	✓		∗	✓	✓	✓	✓	✓	
Totals	22	12	11	15	22	16	13	20	6	4	9	11	10	8	5	10	9	9	23	29	28	24	23	9
% of countries (*n* = 193 total)	11%	6%	6%	8%	11%	8%	7%	10%	3%	2%	5%	6%	5%	4%	3%	5%	5%	5%	12%	15%	15%	12%	12%	5%
% of school meal policies (*n* = 29 total)	78%	36%	38%	52%	76%	55%	45%	69%	21%	14%	31%	38%	34%	28%	17%	34%	31%	31%	79%	100%	97%	83%	79%	31%

Abbreviation: NNS = non-nutritive sweeteners.

**✓***indicates restriction on all foods or beverages.*

**∗** Indicates restriction applied to specific types of food or beverages.

Sixty-nine percent (*n* = 20) of policies applied to all grade levels. All policies applied to school meals in primary schools (100%, *n* = 29) and nearly all in lower secondary schools (97%, *n* = 28), whereas slightly fewer restrictions applied to school meals in upper secondary schools (83%, *n* = 24) and preschools (29%, *n* = 23).

To determine which foods and drinks would not be permitted in the school meal program, the majority of countries used a combination of nutrient thresholds and categorical limitations (72%, *n* = 21). Five countries (17%) restricted foods and drinks only by listing categories not permitted for service, and 3 used only nutrient or ingredient thresholds not specific to categories (10%). Commonly restricted categories included fried foods, candies and desserts, cured meats, and processed snacks.

**FIGURE 1:**
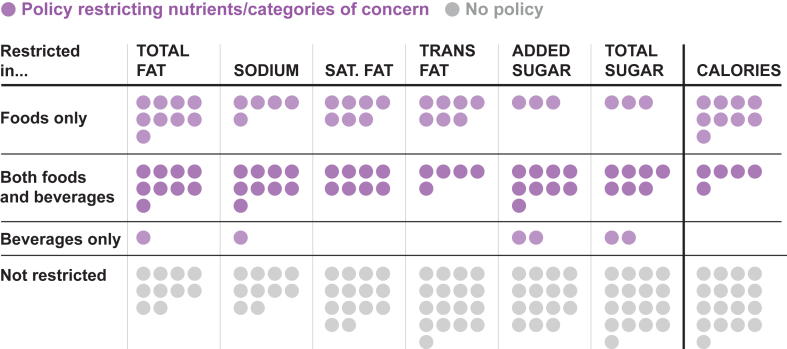
Nutrient content restricted in national school meal policies.

Policies with limits or thresholds for the content of specific nutrients often had different standards for foods and beverages or even for specific categories (Table 2). Among policies with nutrient-specific standards for foods (*n* = 27), the most commonly restricted nutrient was total fat (76% of policies; *n* = 22), followed by sodium (69%, *n* = 20), saturated fat (55%, *n* = 16), added sugar (52%, *n* = 15), then *trans* fat (45%, *n* = 13) (Figure 1). Thirteen countries regulated 5 or more nutrients for food products, and only 1 country (Ukraine) restricted all of these nutrients and calories. Only 11 policies restricted total sugar in foods, and 6 restricted the content of non-nutritive sweeteners. For each nutrient restricted, the majority of policies utilized universal nutrient restrictions rather than limiting content for specific food categories.

Among beverages, the majority of school meal standards used specific nutrient limits (72%, *n* = 21). The most common nutrient content restrictions were on added sugar (38% of school meal policies, *n* = 11), total fat and sodium (both 34%, *n* = 10), and total sugar (31%, *n* = 9), followed by saturated fat (28%, *n* = 8), *trans* fat (17%, *n* = 5), and total calories (14%, *n* = 4).

Five policies completely prohibited serving beverages containing caffeine, and 4 limited caffeine content in specific beverage categories. Six countries had policies prohibiting the provision of any foods or beverages containing non-nutritive sweeteners. Four countries prohibited the use of non-nutritive sweeteners in specific categories (e.g., milk, juices, or certain condiments).

### Monitoring and enforcement

**FIGURE 2:**
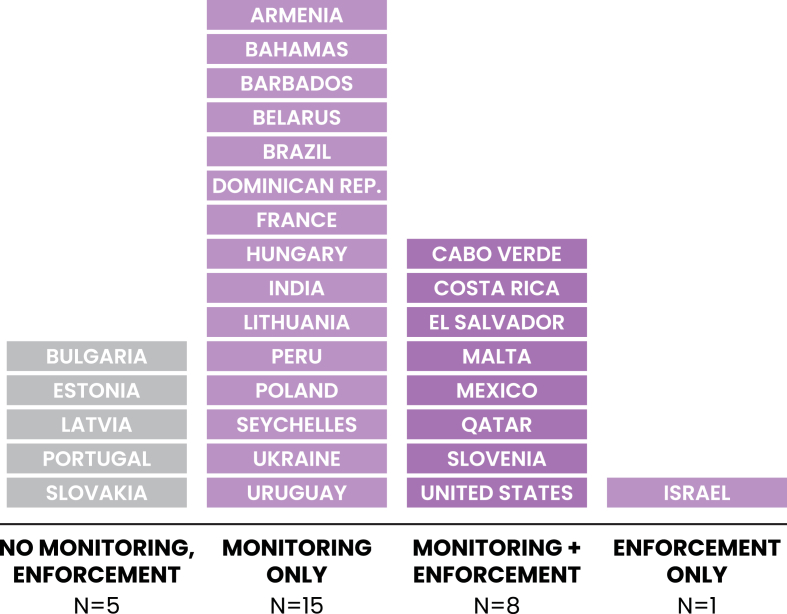
Restrictive school meal policies’ inclusion of provisions for monitoring and enforcement.

Twenty-three countries (79% of policies) included monitoring language in their school meal regulation, whereas only 31% (*n* = 9) included explicit enforcement language (Figure 2). Policies that included provisions for monitoring often named a committee external to the school system responsible for periodically reviewing school meal quality and compliance with regulations. Enforcement mechanisms included monetary fines for noncompliance and/or employment repercussions for those managing and serving school meals. Over half of policies with monitoring provisions did not specify enforcement mechanisms.

### Policies by country income classification and geographic region

#### Income classification

**FIGURE 3:**
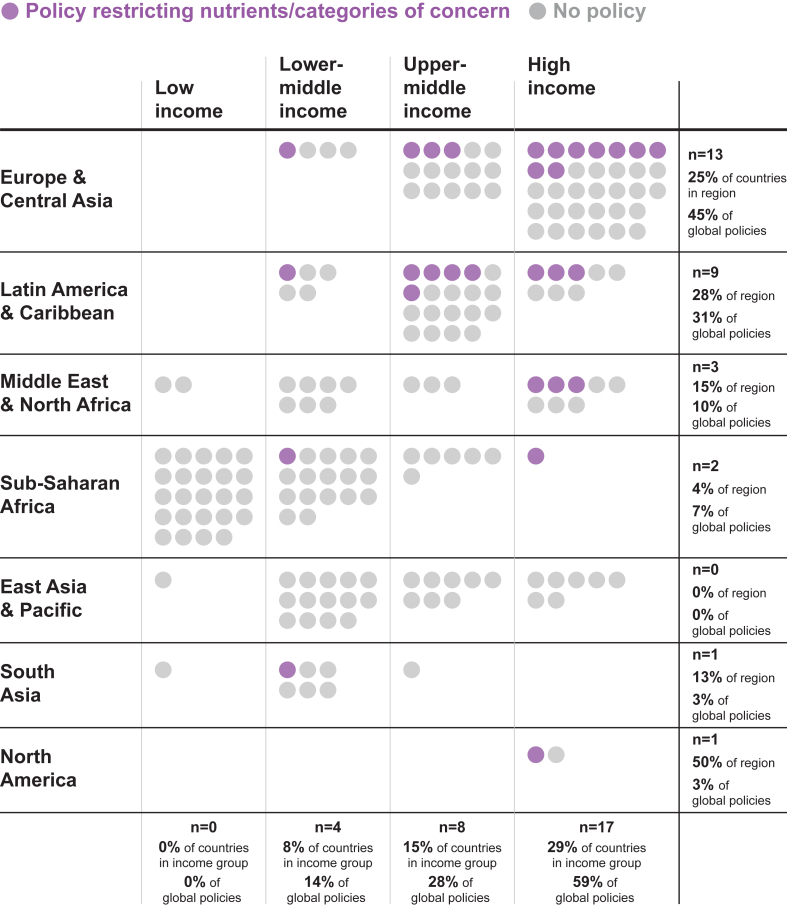
Countries with national, mandatory policies restricting nutrients or categories of concern in school meals by world region and income level.

School meal policies restricting foods or nutrients of concern were most prevalent among HICs and decreased with each income level (Figure 3). Fifty-nine percent (*n* = 17) of policies were found in HICs, 28% (*n* = 8) in upper-middle-income countries (UMICs), and 14% (*n* = 4) in LMICs. No policies restricting the foods and beverages served through a national school meal program were found in LIC.

Among the 59 HICs, 29% had a school meal policy with nutritional restrictions (*n* = 17). Of 52 UMICs, 15% of countries were found to have policies (*n* = 8). Of 53 LMICs, 8% were found to have policies (*n* = 4), and none of the 28 LICs had this type of policy.

The proportion of policies that used nutrient content limits was similar across country income groups: 76% (*n* = 13) of policies in HICs restricted content for 1 or more nutrients, as did 75% (*n* = 3) of LMIC policies and 63% (*n* = 5) of UMIC policies. The majority of policies in all regions restricted service of entire categories of foods in school meals (often in addition to applying nutritional criteria), with 88% (*n* = 7) of policies in UMICs, 75% (*n* = 3) of policies in LMICs, and 71% (*n* = 12) of policies in HICs including ≥1 restricted category.

Conversely, the inclusion of monitoring language was most common among LMICs. All policies in LMICs (100%, *n* = 4) included monitoring language, followed by 88% of UMICs (*n* = 7) and 71% of HICs (*n* = 12). The inclusion of enforcement criteria was less common among all income groups, with 50% (*n* = 2) of policies in LMICs, 29% (*n* = 5) in HICs, and 25% (*n* = 2) in UMICs, including enforcement language.

LMICs had the highest proportion of regulations that covered preschools with their school meal policies (100%, *n* = 4) compared to UMICs (75%, *n* = 6) and HICs (71%, *n* = 12). This pattern was similar for coverage of secondary schools: All 4 LMIC policies covered upper secondary schools (100%) compared with 82% of HIC policies (*n* = 14) and 75% of UMIC policies (*n* = 6). The distribution of policy coverage in primary and lower secondary grades was similar across all country income groups, with almost 100% coverage.

#### Geographic region

The Europe and Central Asia region had the most policies (*n* = 13), followed by Latin America and the Caribbean (*n* = 9). The East Asia and Pacific region had no policies meeting inclusion criteria. All 9 policies in the Latin America and Caribbean region included monitoring requirements, but only 3 specified enforcement mechanisms. The few policies found in Sub-Saharan Africa, North America, and South Asia all included monitoring (*n* = 2, *n* = 1, and *n* = 1, respectively), but only 2 of those specified enforcement mechanisms (Qatar and the United States).

Countries in Latin America also included more comprehensive nutrient profiling in their policies. Of the 13 policies that regulated 5 or more nutrients, 6 were in the Latin America and Caribbean region, 4 in Europe and Central Asia, 2 in the Middle East and North Africa, and 1 in Sub-Saharan Africa.

The distribution of policies limiting products by category was similar across geographical regions. None of the 3 policies in the Middle East and North Africa region or North America regions applied to preschools. Policies in all other geographic regions most often applied to kindergarten through upper secondary grades, with all but 3 policies also covering preschools and all but 5 policies across regions covering secondary schools.

#### Health promotion

Although this article focused on policies restricting unhealthy foods in school meals, we also collected some data on whether the included policies also had provisions to promote or require healthy foods in school meals. (This would not include countries with policies focused solely on health-promoting foods without any restriction on unhealthy items). All 29 restrictive school meal policies also included regulations ensuring or requiring the service of nutrient-dense foods. Common criteria for promoting healthy foods included requirements for the number of servings of fruits and vegetables (daily or weekly), serving a variety of food groups in each meal, and providing plain, clean water.

## Discussion

Our scoping review found that national-level, mandatory policies to limit the service of foods and beverages, nutrients, or ingredients of health concern through government-sponsored school meal programs are not common worldwide: Only 15% of countries (*n* = 29) were found to have such a policy. Given the scope of this review, we recognize that our findings do not reflect a number of countries that have statutory regulations pertaining to the nutritional quality of foods served in schools but lack a formal, national school meal program. Nevertheless, the low prevalence of policies meeting inclusion criteria for this review does suggest that many schoolchildren worldwide are likely not receiving school meals that meet their dietary requirements and are provided in a consistent and reliable manner.

Notably, none of the policies that met our inclusion criteria were found in LICs. Included policies were most common among HICs and decreased in prevalence as income designation decreased. We also observed this pattern in our companion article assessing the presence of competitive food restrictions and marketing restrictions in the school environment [31]. This trend may result from insufficient funding or infrastructure to implement school meal programs on a national level or from a greater focus on providing adequate calories (rather than restricting unhealthy foods or nutrients) in LIC school meal programs. Another reason for this trend could be differing practices around school mealtimes. For example, in countries where children go home to eat lunch, a national school meal program may not be necessary or desired. Partnership with and funding from nongovernmental donors could also impact the quality of school meals in some settings, but such arrangements were outside the scope of our review and were not included in this analysis. Further research is needed on the prevalence, nutritional quality, and adequacy of school meal programs in LICs. Such population-level nutritional interventions can be particularly impactful in LICs that face a double burden of malnutrition — rising levels of overweight and obesity alongside high prevalence of stunting, wasting, and micronutrient deficiencies — particularly as these countries have lower resources to treat and manage noncommunicable disease that can arise from poor diets.

The regions of Europe and Central Asia, and Latin America and the Caribbean had the highest absolute number and greatest global concentration of school meal policies in the world. Countries in the Latin America and Caribbean region, in particular, have been developing and implementing innovative and robust school food environment policies to address rising obesity rates, including regulating school meals alongside restrictions on competitive foods and marketing [31]. Policies in the Latin America and Caribbean region restrict the content of a greater number of nutrients compared to other regions, likely due to widespread use of the Pan American Health Organization (PAHO) nutrient profile for their school food policies [76].

### Highlighting impactful policies

Several policies stand out for their robust nutrition requirements, successful implementation, and positive impact on child nutrition and health. Further analysis of the best practices within these policies can provide guidance for other countries to implement or modify their school nutrition standards. As these approaches have also been found effective at improving dietary quality, understanding their designs can enhance the effectiveness of new or existing policies.

Brazil has been recognized for its innovative national school meal program, PNAE, that provides free meals to all students in all grade levels [43,77]. Under a 2009 law (amended in 2013) and an additional 2020 resolution [42,43,78], PNAE regulations ban serving drinks low in nutritional value, such as sodas and carbonated beverages, as well as foods high in sodium and saturated fat, such as sausages or other canned foods. PNAE regulations also offer suggestions for meal composition with regard to the proportion of added sugars, total fat, saturated fat, *trans* fat, and sodium. In addition to these restrictions, schools must serve ≥3 fruits and vegetables per week, and 30% of foods procured must be purchased from family-owned farms in Brazil. In addition, 75% of all food is fresh or minimally processed, no >5% is minimally processed culinary ingredients, and no >20% may be ultra-processed. Consumption of school meals in Brazil has been associated with increased intake of nutritious foods and reduced prevalence of overweight and obesity among youth and adolescents [13,14,79]. A national evaluation of the PNAE’s combined regulations is currently underway.

In the United States, the Healthy, Hunger-Free Kids Act of 2010 modified the requirements of the national school lunch and school breakfast program by implementing limits on calories, saturated fat, *trans* fat, and sodium content across all foods served in school meals, which are monitored by state agencies to ensure compliance [80]. The United States also places some restrictions on in-school marketing and competitive food sales for less-healthy foods, as discussed in this article’s companion review [31]. These strengthened standards resulted in significant improvements in the nutritional quality of school meals and better alignment with the United States dietary guidelines [81]. Improvements included offering more servings of fruits and vegetables, offering a wider variety of foods, and regulating portion sizes of foods offered in school meals [82].

Despite these successes, many of the foods served in United States schools remain ultra-processed [[83], [84], [85]]. In the United States and worldwide, the many elevated health risks associated with the high consumption of ultra-processed products — even independent of their nutritional content — suggest that policymakers should restrict the service of foods in schools based on degree of processing in addition to nutritional profile and should maximize provision of whole or minimally processed foods [3,86]. This will require commitments to fund the procurement of healthier foods and staffing for more intensive food preparation, as well as ensuring schools have adequate facilities and equipment for food preparation and storage.

### School meal challenges

Adoption of school meal regulations does not guarantee that all students will reap the benefits of the policy, as there are multiple barriers to school-level implementation and student-level meal access. Country-level evaluations and scoping reviews have found that schools can struggle to implement meal standards. For example, an evaluation of Slovenia’s school meals program found that school characteristics such as size, location, and financial resources can play an important role in effective implementation, with larger schools in higher-income districts having better menu quality [87]. For schools with fewer resources, higher prices and limited availability of healthier foods can present barriers [88]. This is also impacted by a country’s funding and enforcement structures for the school meal program, as different countries provide varying levels of support for the cost of meal inputs, labor time, and kitchen infrastructure. Before the United States mandated compliance with nutrition standards and offered additional training and incentives to meet them, only 22.7% of middle school and 15.3% of high school students attended schools with compliant nutrition environments, but compliance increased over time and by 2014–2015, most menus nationwide met daily requirements for types and amounts of food offered at lunch, with 93% meeting the saturated fat limit and 72% compliant with the sodium limit [89,90]. As of 2020, 99% of national school lunch program schools were meeting nutrition standards [91].

In schools that do not offer free meals to all students, meal costs can impede participation and prevent lower-income students from receiving the benefits of meals that meet dietary guidelines. Some schools and parents also report low meal participation rates during the initial implementation of nutrition standards because healthier foods may have lower initial acceptability to students [92,93], but uptake improves over time as students grow accustomed to new items [20, 82,[94], [95], [96], [97]]. Coupling meal changes with taste tests, nutrition education, longer meal times, and limits on competitive foods has been shown to mitigate this issue [98,99].

Lastly, the reach of school meal policies is also inherently limited to a certain population and setting. Unless regulations cover facilities beyond the school setting, the reach of benefits from school meal standards is limited to schoolchildren, which does not accommodate children aged 0–4 who are not in preschool (who are in a critical development stage) or any child of school age who is outside of the school system. This may be a growing problem due to rising levels of absenteeism after the COVID-19 pandemic [100,101].

Without complementary, population-wide regulations to limit demand for and access to unhealthy foods, such as front-of-package labeling, marketing restrictions, or nutrition-related fiscal policies, schoolchildren can also be exposed to unhealthy foods at home and in their communities, especially when school is not in session. The health and wellbeing impacts of school meals depend on the proportion of food children actually consume at school. All of these factors contribute to the difficulty of assessing the population health impact of school meal regulations, which can further impact their funding and support.

### Health-promoting environment

Most countries’ restrictive policies also include health-promoting measures (e.g., minimum requirements for fruits and vegetables or whole grains). In addition to the types of foods regulated, several policies also promote local procurement of foods served in schools to increase quality and support the local economy. For example, in Latvia, schools prioritize purchases within 300 km of each school for sourcing fruits, vegetables, and roots/tubers used in the school milk and fruit program. Other school meal programs promote purchasing sustainably grown and/or organic foods. In France, for example, 50% of meals served in schools must be sustainable food products, 20% of which must be organic. These provisions were more commonly found among countries in Europe and Central Asia.

Although extensive data on health-promoting school meal measures was not captured in this review, the high prevalence of healthy food requirements within restrictive policies indicates that many policies are taking a multi-pronged approach to improve the school food environment. Further research is needed to explore the impact of such dual-intention policies.

### Monitoring and enforcement

Our study finds room for improvement in the inclusion of monitoring and enforcement mechanisms in school meal policies, as 79% of countries’ policies included monitoring language, and only 31% included enforcement language. Even in countries with policies that did include monitoring, and enforcement mechanisms, there is evidence of schools’ noncompliance. For example, Costa Rica’s school meal policy (which limits specific nutrients among certain food categories and prohibits serving deep-fried foods) requires monitoring by an external health and nutrition committee and enforcement via escalation and removal of duties for staff who fail to adhere to the policy. However, a study that conducted semi-structured interviews and site observations in Costa Rican schools found that restricted foods were still readily available in the school environment [97]. Further research is needed to understand barriers to implementation and compliance and how to devise policy language to ensure that regulations can be implemented as intended.

### Limitations

This review had several key limitations. Although we conducted an extensive search to source primary policy documents for all regulations included in this review, it is possible that some documentation was missed due to a lack of availability online or language barriers. Despite attempts to prevent or mitigate such errors, accurate interpretation of policies may have been impacted due to translation errors or ambiguous policy language, despite attempts to prevent or mitigate such errors.

In addition, our focus on policies regulating nutritional content, specifically in nationwide, government-sponsored school meal programs, led to the exclusion of some policies with more broadly applied nutritional standards for foods that are essentially consumed as meals in schools but are not part of a formal meal program. For example, Ecuador has compulsory nutrition standards for foods served or sold in schools (whether as snacks or a meal) but has not had a formal national school lunch program since 2013 [102,103]. Policies excluded for this reason still serve an important role — especially in countries that do not yet have national school meal programs — in ensuring the availability of high-quality, healthy food and beverage choices and keeping less-healthy foods out of schools. We included these in our companion scoping review on competitive food and marketing policies in schools.

We also limited the focus of this review to only national-level mandatory policies and thus did not capture policies implemented by smaller jurisdictions (e.g., states, provinces, and municipalities). In this review, we found some countries with relatively comprehensive national coverage that were not mandatory or were implemented at state or local levels. For example, Australia, Canada, and the United Kingdom do have a national-level school meal program, but policies at state, province, or territory levels are reportedly well-implemented and, in effect, achieve national coverage [[104], [105], [106], [107], [108], [109]]. Additionally, some countries have comprehensive policies that are structured as voluntary guidelines yet have high reported compliance, such as in Finland and Japan [[110], [111], [112], [113], [114]]. These seem to be exceptions to the norm, as evidence shows voluntary policies are generally not an effective approach for shaping and protecting a healthy food environment [[110], [111], [112], [113], [114], [115]]. Future research should investigate the global prevalence of mandatory school meal policy implementation at sub-national levels.

Finally, there are many factors that contribute to the effectiveness and reach of school meal programs, including pricing policies, procurement and equipment challenges, time and budgetary constraints, food quality, student preferences and consumption patterns, school attendance, and policy enforcement, among others. These factors greatly impact the ability to draw conclusions about the effectiveness of school meal policy designs without additional research. Future research should examine barriers to policy adoption and implementation in addition to evaluating whether these interventions have the desired impacts on dietary quality and adequacy, changes in overall dietary patterns among children, and ultimately, improvements in population health and wellbeing. Future studies should also build on this review by examining the global landscape for related school food environment policies involving procurement, nudging interventions to promote healthy food behavior, and pricing [17]. Competitive food and marketing restrictions are examined in a companion scoping review [31].

We anticipate the global landscape of national school meal policies will expand and improve in the coming years, as work is already underway in many places to strengthen school feeding programs. For example, at the 2021 United Nations Food Systems Summit, 82 governments joined in launching the school meals coalition, which aims to ensure that “by 2030, every child can receive a healthy, nutritious daily meal in school” [116]. As of 2023, another 16 countries had joined, and 47 countries had defined national commitments to achieve that goal [117].

In addition, momentum around the adoption of food-related policies to improve population health and combat rising noncommunicable disease rates has built in recent years, with increasing worldwide implementation of front-of-package labeling laws, taxes on unhealthy foods and beverages, marketing restrictions for these products, and more. Increased focus on school food environment policies has been part of this movement: these policies are supported by world health leaders as the key to addressing all forms of childhood malnutrition and establishing a lifelong trajectory for healthy diets and good health [[118], [119], [120], [121]]. These organizations provide implementation guidance to countries interested in adopting new or strengthening existing policies related to the school food environment [17,116,122,123].

In conclusion, providing school meals that adequately nourish growing children and adolescents is a critical policy imperative. Our review finds that very few countries currently leverage policies to limit the presence of foods and beverages that can harm health in a national school meal program. More widespread adoption of well-monitored and well-enforced school meal policies is needed, as well as more research on which policy components or designs are most impactful. This review focuses on policies that limit nutrients, ingredients, or food categories of health concern served in school meals, but many policies include or prioritize provisions to increase students’ intake of healthy foods such as minimally processed vegetables, fruits, whole grains, legumes, and water. Both of these components are needed to provide students with adequate nourishment and establish healthy lifelong eating preferences and habits. School meal standards can play a particularly important role in LMICs, where energy-dense, ultra-processed foods and drinks high in sugar, sodium, and saturated fat have not yet come to dominate children’s diets [2]. Preventing these from becoming normalized in school settings could preempt the displacement of more health-building foods and limit increases in obesity prevalence, whereas also reducing stunting, wasting, and micronutrient deficiencies in areas facing a double burden of malnutrition.

Future research should evaluate the implementation and efficacy of national school meal policies for improving health, as well as their combined impact with complementary policies focused on the broader food environment [31]. In all countries, strong school meal policies have the potential to improve dietary quality and lifelong health for all children attending school, regardless of household resources.

## Author contributions

The authors’ responsibilities were as follows– BMP, EAB: conceptualized the study; EAB, MP, LST, FRDC: drafted the codebook; GC, KM: piloted the codebook and conducted data collection and coding; MP, GC, KM: conducted data analysis; all authors contributed to the writing and editing of the manuscript; and all authors: read and approved the final manuscript.

## Funding

This study was financially supported by Bloomberg Philanthropies, who had no involvement or restrictions regarding this submission for publication.

### Data availability

Data described in this manuscript will be made available upon request.

## Conflict of interest

The authors declare the following financial/personal relationships which may be considered as potential competing interests: Barry Popkin reports a relationship with National Institutes of Health that includes: funding grants. Barry Popking reports a relationship with World Bank that includes: consulting or advisory. Barry Popkin reports a relationship with Resolve to Save Lives that includes: consulting or advisory. Francesca Dillman Carpentier reports a relationship with World Bank that includes: consulting or advisory. Lindsey Smith Taillie reports a relationship with National Institutes of Health that includes: funding grants. Lindsey Smith Taillie reports a relationship with Resolve to Save Lives that includes: consulting or advisory. Lindsey Smith Taillie reports a relationship with Global Alliance for Improved Nutrition that includes: consulting or advisory. If there are other authors, they declare that they have no known competing financial interests or personal relationships that could have appeared to influence the work in this paper.
