# Supplementary material for: National Policies to Limit Nutrients, Ingredients, or Categories of Concern in School Meals: A Global Scoping Review
Source: Curr Dev Nutr. 2024 Sep 19;8(10):104456. doi: 10.1016/j.cdnut.2024.104456 (PMC11490762; doi:10.1016/j.cdnut.2024.104456)
Supplement: Multimedia component 1 [file mmc1.pdf]

National policies to limit nutrients, ingredients, or categories of concern in school meals: a global scoping review

Emily A. Busey, Grace Chamberlin, Kayla Mardin, Michelle Perry, Lindsey Smith Taillie, Francesca R. Dillman Carpentier, Barry M. Popkin

Supplemental Materials

Supplemental Table 1. Countries included in study sample (n=193), World Bank region and income classifications, and policy status 2

Supplemental Table 2. Inclusion and exclusion criteria 8

Supplemental Figure 1. Search strategy 9

Supplemental Text 1. Sample search strategy 10

Supplemental Table 3. Codebook 11

References 18

Supplemental Table 1. Countries included in study sample (n=193), World Bank region and income classifications, and policy status

Country[a]	World Bank region	World Bank income classification	National, mandatory policy restricting service of foods/beverages, nutrients, or ingredients of health concern in school meals
**Afghanistan**	South Asia	Low Income	
**Albania**	Europe & Central Asia	Upper Middle Income	
**Algeria**	Middle East & North Africa	Lower Middle Income	
**Andorra**	Europe & Central Asia	High Income	
**Angola**	Sub-Saharan Africa	Lower Middle Income	
**Antigua and Barbuda**	Latin America & Caribbean	High Income	
**Argentina**	Latin America & Caribbean	Upper Middle Income	
**Armenia**	Europe & Central Asia	Upper Middle Income	✓
**Australia**	East Asia & Pacific	High Income	
**Austria**	Europe & Central Asia	High Income	
**Azerbaijan**	Europe & Central Asia	Upper Middle Income	
**Bahamas**	Latin America & Caribbean	High Income	✓
**Bahrain**	Middle East & North Africa	High Income	
**Bangladesh**	South Asia	Lower Middle Income	
**Barbados**	Latin America & Caribbean	High Income	✓
**Belarus**	Europe & Central Asia	Upper Middle Income	✓
**Belgium**	Europe & Central Asia	High Income	
**Belize**	Latin America & Caribbean	Upper Middle Income	
**Benin**	Sub-Saharan Africa	Lower Middle Income	
**Bhutan**	South Asia	Lower Middle Income	
**Bosnia and Herzegovina***	Europe & Central Asia	Upper Middle Income	
**Botswana**	Sub-Saharan Africa	Upper Middle Income	
**Brazil**	Latin America & Caribbean	Upper Middle Income	✓
**Brunei Darussalam**	East Asia & Pacific	High Income	
**Bulgaria***	Europe & Central Asia	Upper Middle Income	✓
**Burkina Faso**	Sub-Saharan Africa	Low Income	
**Burundi**	Sub-Saharan Africa	Low Income	
**Cabo Verde**	Sub-Saharan Africa	Lower Middle Income	✓
**Cambodia**	East Asia & Pacific	Lower Middle Income	
**Cameroon**	Sub-Saharan Africa	Lower Middle Income	
**Canada**	North America	High Income	

Country[a]	World Bank region	World Bank income classification	National, mandatory policy restricting service of foods/beverages, nutrients, or ingredients of health concern in school meals
**Central African Republic**	Sub-Saharan Africa	Low Income	
**Chad**	Sub-Saharan Africa	Low Income	
**Chile**	Latin America & Caribbean	High Income	
**China**	East Asia & Pacific	Upper Middle Income	
**Colombia**	Latin America & Caribbean	Upper Middle Income	
**Comoros**	Sub-Saharan Africa	Lower Middle Income	
**Congo**	Sub-Saharan Africa	Lower Middle Income	
**Costa Rica**	Latin America & Caribbean	Upper Middle Income	✓
**Côte d'Ivoire**	Sub-Saharan Africa	Lower Middle Income	
**Croatia**	Europe & Central Asia	High Income	
**Cuba***	Latin America & Caribbean	Upper Middle Income	
**Cyprus***	Europe & Central Asia	High Income	
**Czechia**	Europe & Central Asia	High Income	
**Democratic People's Republic of Korea**	East Asia & Pacific	Low Income	
**Democratic Republic of the Congo**	Sub-Saharan Africa	Low Income	
**Denmark**	Europe & Central Asia	High Income	
**Djibouti**	Middle East & North Africa	Lower Middle Income	
**Dominica**	Latin America & Caribbean	Upper Middle Income	
**Dominican Republic**	Latin America & Caribbean	Upper Middle Income	✓
**Ecuador**	Latin America & Caribbean	Upper Middle Income	
**Egypt**	Middle East & North Africa	Lower Middle Income	
**El Salvador**	Latin America & Caribbean	Lower Middle Income	✓
**Equatorial Guinea**	Sub-Saharan Africa	Upper Middle Income	
**Eritrea**	Sub-Saharan Africa	Low Income	
**Estonia**	Europe & Central Asia	High Income	✓
**Eswatini**	Sub-Saharan Africa	Lower Middle Income	
**Ethiopia**	Sub-Saharan Africa	Low Income	
**Federated States of Micronesia**	East Asia & Pacific	Lower Middle Income	
**Fiji**	East Asia & Pacific	Upper Middle Income	
**Finland***	Europe & Central Asia	High Income	
**France**	Europe & Central Asia	High Income	✓
**Gabon**	Sub-Saharan Africa	Upper Middle Income	

Country[a]	World Bank region	World Bank income classification	National, mandatory policy restricting service of foods/beverages, nutrients, or ingredients of health concern in school meals
**Gambia**	Sub-Saharan Africa	Low Income	
**Georgia**	Europe & Central Asia	Upper Middle Income	
**Germany**	Europe & Central Asia	High Income	
**Ghana**	Sub-Saharan Africa	Lower Middle Income	
**Greece**	Europe & Central Asia	High Income	
**Grenada***	Latin America & Caribbean	Upper Middle Income	
**Guatemala**	Latin America & Caribbean	Upper Middle Income	
**Guinea**	Sub-Saharan Africa	Low Income	
**Guinea-Bissau**	Sub-Saharan Africa	Low Income	
**Guyana**	Latin America & Caribbean	Upper Middle Income	
**Haiti**	Latin America & Caribbean	Lower Middle Income	
**Honduras***	Latin America & Caribbean	Lower Middle Income	
**Hungary**	Europe & Central Asia	High Income	✓
**Iceland**	Europe & Central Asia	High Income	
**India**	South Asia	Lower Middle Income	✓
**Indonesia**	East Asia & Pacific	Lower Middle Income	
**Iraq**	Middle East & North Africa	Upper Middle Income	
**Ireland**	Europe & Central Asia	High Income	
**Islamic Republic of Iran**	Middle East & North Africa	Lower Middle Income	
**Israel***	Middle East & North Africa	High Income	✓
**Italy**	Europe & Central Asia	High Income	
**Jamaica***	Latin America & Caribbean	Upper Middle Income	
**Japan***	East Asia & Pacific	High Income	
**Jordan**	Middle East & North Africa	Upper Middle Income	
**Kazakhstan**	Europe & Central Asia	Upper Middle Income	
**Kenya**	Sub-Saharan Africa	Lower Middle Income	
**Kiribati**	East Asia & Pacific	Lower Middle Income	
**Kuwait***	Middle East & North Africa	High Income	
**Kyrgyzstan**	Europe & Central Asia	Lower Middle Income	
**Lao People's Democratic Republic**	East Asia & Pacific	Lower Middle Income	
**Latvia**	Europe & Central Asia	High Income	✓
**Lebanon**	Middle East & North Africa	Lower Middle Income	
**Lesotho**	Sub-Saharan Africa	Lower Middle Income	
**Liberia**	Sub-Saharan Africa	Low Income	

Country[a]	World Bank region	World Bank income classification	National, mandatory policy restricting service of foods/beverages, nutrients, or ingredients of health concern in school meals
**Libya**	Middle East & North Africa	Upper Middle Income	
**Liechtenstein**	Europe & Central Asia	High Income	
**Lithuania**	Europe & Central Asia	High Income	✓
**Luxembourg**	Europe & Central Asia	High Income	
**Madagascar**	Sub-Saharan Africa	Low Income	
**Malawi**	Sub-Saharan Africa	Low Income	
**Malaysia**	East Asia & Pacific	Upper Middle Income	
**Maldives**	South Asia	Upper Middle Income	
**Mali**	Sub-Saharan Africa	Low Income	
**Malta**	Middle East & North Africa	High Income	✓
**Marshall Islands**	East Asia & Pacific	Upper Middle Income	
**Mauritania**	Sub-Saharan Africa	Lower Middle Income	
**Mauritius**	Sub-Saharan Africa	Upper Middle Income	
**Mexico**	Latin America & Caribbean	Upper Middle Income	✓
**Monaco**	Europe & Central Asia	High Income	
**Mongolia**	East Asia & Pacific	Lower Middle Income	
**Montenegro**	Europe & Central Asia	Upper Middle Income	
**Morocco**	Middle East & North Africa	Lower Middle Income	
**Mozambique**	Sub-Saharan Africa	Low Income	
**Myanmar**	East Asia & Pacific	Lower Middle Income	
**Namibia**	Sub-Saharan Africa	Upper Middle Income	
**Nauru**	East Asia & Pacific	High Income	
**Nepal***	South Asia	Lower Middle Income	
**Netherlands**	Europe & Central Asia	High Income	
**New Zealand**	East Asia & Pacific	High Income	
**Nicaragua**	Latin America & Caribbean	Lower Middle Income	
**Niger**	Sub-Saharan Africa	Low Income	
**Nigeria**	Sub-Saharan Africa	Lower Middle Income	
**North Macedonia**	Europe & Central Asia	Upper Middle Income	
**Norway**	Europe & Central Asia	High Income	
**Oman***	Middle East & North Africa	High Income	
**Pakistan**	South Asia	Lower Middle Income	
**Palau***	East Asia & Pacific	Upper Middle Income	
**Panama**	Latin America & Caribbean	High Income	
**Papua New Guinea**	East Asia & Pacific	Lower Middle Income	

Country[a]	World Bank region	World Bank income classification	National, mandatory policy restricting service of foods/beverages, nutrients, or ingredients of health concern in school meals
**Paraguay**	Latin America & Caribbean	Upper Middle Income	
**Peru**	Latin America & Caribbean	Upper Middle Income	✓
**Philippines**	East Asia & Pacific	Lower Middle Income	
**Plurinational State of Bolivia**	Latin America & Caribbean	Lower Middle Income	
**Poland***	Europe & Central Asia	High Income	✓
**Portugal**	Europe & Central Asia	High Income	✓
**Qatar**	Middle East & North Africa	High Income	✓
**Republic of Korea***	East Asia & Pacific	High Income	
**Republic of Moldova***	Europe & Central Asia	Upper Middle Income	
**Romania**	Europe & Central Asia	High Income	
**Russian Federation**	Europe & Central Asia	Upper Middle Income	
**Rwanda**	Sub-Saharan Africa	Low Income	
**Saint Kitts and Nevis**	Latin America & Caribbean	High Income	
**Saint Lucia**	Latin America & Caribbean	Upper Middle Income	
**Saint Vincent and the Grenadines**	Latin America & Caribbean	Upper Middle Income	
**Samoa***	East Asia & Pacific	Lower Middle Income	
**San Marino**	Europe & Central Asia	High Income	
**São Tomé and Príncipe**	Sub-Saharan Africa	Lower Middle Income	
**Saudi Arabia**	Middle East & North Africa	High Income	
**Senegal**	Sub-Saharan Africa	Lower Middle Income	
**Serbia**	Europe & Central Asia	Upper Middle Income	
**Seychelles**	Sub-Saharan Africa	High Income	✓
**Sierra Leone**	Sub-Saharan Africa	Low Income	
**Singapore**	East Asia & Pacific	High Income	
**Slovakia**	Europe & Central Asia	High Income	✓
**Slovenia**	Europe & Central Asia	High Income	✓
**Solomon Islands**	East Asia & Pacific	Lower Middle Income	
**Somalia**	Sub-Saharan Africa	Low Income	
**South Africa**	Sub-Saharan Africa	Upper Middle Income	
**South Sudan**	Sub-Saharan Africa	Low Income	
**Spain**	Europe & Central Asia	High Income	
**Sri Lanka**	South Asia	Lower Middle Income	
**Sudan**	Sub-Saharan Africa	Low Income	

Country[a]	World Bank region	World Bank income classification	National, mandatory policy restricting service of foods/beverages, nutrients, or ingredients of health concern in school meals
**Suriname**	Latin America & Caribbean	Upper Middle Income	
**Sweden**	Europe & Central Asia	High Income	
**Switzerland**	Europe & Central Asia	High Income	
**Syrian Arab Republic**	Middle East & North Africa	Low Income	
**Tajikistan**	Europe & Central Asia	Lower Middle Income	
**Thailand**	East Asia & Pacific	Upper Middle Income	
**Timor-Leste**	East Asia & Pacific	Lower Middle Income	
**Togo**	Sub-Saharan Africa	Low Income	
**Tonga***	East Asia & Pacific	Upper Middle Income	
**Trinidad and Tobago**	Latin America & Caribbean	High Income	
**Tunisia**	Middle East & North Africa	Lower Middle Income	
**Türkiye**	Europe & Central Asia	Upper Middle Income	
**Turkmenistan**	Europe & Central Asia	Upper Middle Income	
**Tuvalu**	East Asia & Pacific	Upper Middle Income	
**Uganda**	Sub-Saharan Africa	Low Income	
**Ukraine***	Europe & Central Asia	Lower Middle Income	✓
**United Arab Emirates**	Middle East & North Africa	High Income	
**United Kingdom**	Europe & Central Asia	High Income	
**United Republic of Tanzania**	Sub-Saharan Africa	Lower Middle Income	
**United States of America**	North America	High Income	✓
**Uruguay**	Latin America & Caribbean	High Income	✓
**Uzbekistan**	Europe & Central Asia	Lower Middle Income	
**Vanuatu**	East Asia & Pacific	Lower Middle Income	
**Venezuela**	Latin America & Caribbean	N/A	
**Viet Nam**	East Asia & Pacific	Lower Middle Income	
**Yemen**	Middle East & North Africa	Low Income	
**Zambia**	Sub-Saharan Africa	Low Income	
**Zimbabwe**	Sub-Saharan Africa	Lower Middle Income	

[a] 2022-2023 United Nations designations
* Query sent to in-country contacts for clarification on policy interpretation.

Supplemental Table 2. Inclusion and exclusion criteria

Inclusion	Exclusion	Exclusion example
Has a **national school meal program**	Does not have a **national school meal program**	*Though Fiji has School Food and Canteen Policy, the country lacked a national school meal program as of the data collection for this paper.*[1]
Has a policy establishing **compulsory nutrition standards** for the school meal program	Has **voluntary nutrition guidelines** or **no nutrition standards** for the school meal program	*Finland has provided free school meals nationwide to preschoolers through secondary school since the 1940s; The National Nutrition Council develops and coordinates voluntary nutrition guidelines for these meals, but the guidelines are not compulsory.*[2]
Nutrition standards **restrict or limit** provision of food categories, nutrients, or ingredients of health concern in school meals	Nutrition standards **do not restrict or limit** provision of food categories, nutrients, or ingredients of health concern in school meals	*The Maldives has a "Health Promoting Schools" policy focused on encouraging healthy foods in schools without restricting service of less-healthy foods.*[3]
Policy is **mandatory**	Policy is **voluntary** (e.g., non-binding guidelines recommendations)	*Japan has widely implemented a school meal program that follows nutrition guidelines which are not mandatory for all schools.*[4–7] *Similarly, Finland has a national school meal program, but school meal nutrition guidelines are voluntary.*[8]
Policy is implemented at the **national level**	Policy is implemented at a **sub-national level** (e.g., province, state, city, territory, district)	*Australia has school nutrition standards implemented at the state/territory level.*[9]

Supplemental Figure 1. Search Strategy

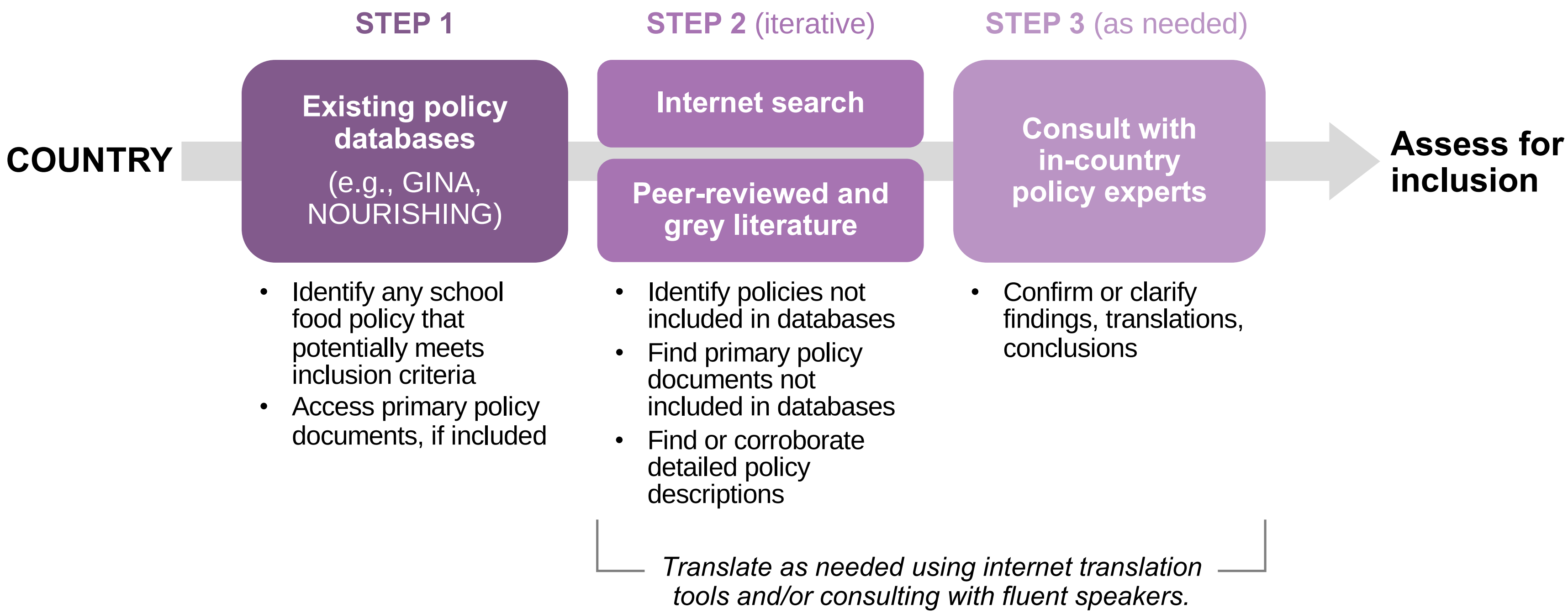

Supplemental Text 1. Sample internet search strategy

Example country: United States of America

Search strategy: We first searched for and identified relevant policies included in the World Health Organization's GINA database (since renamed GIFNA)[10] and World Cancer Research Fund International's NOURISHING database.[11] Next, we searched Google and PubMed using the following search terms to capture any missing, updated, or supplemental policy information and identify primary policy documentation: ["USA" OR "America" OR "United States] AND ["school meals" OR "school food" OR "national school lunch program"] AND ["policy" OR "regulations" OR "requirements" OR "standards"]. This search strategy, as well as the GINA and NOURISHING databases, led us to the United States' federal register to read and code the "Nutrition Standards for All Foods Sold in School as Required by the Healthy, Hunger-Free Kids Act of 2010"[12] as well as regulations and memos regarding the implementation of the school nutrition standards.[13,14] Additionally, the search terms led researchers to peer-reviewed articles assessing the United States' school meal program, which contained additional information and links to original policy documents for data substantiation.[15,16]

Supplemental Table 3. Codebook

Variable #	Variable/ Field Name	Form Name	Section Header	Field Label	Field Type	Choices, Calculations, OR Slider Labels	Skip Logic
1	record_id	Regulations		Record ID<br><br>**Country Name*	text	[open field]	
2	data_entry	Regulations		Person performing country search/data input	dropdown	1, Kayla Mardin<br>2, Grace Chamberlin<br>3, Emily Busey<br>4, Michelle Perry	
3	data_dt	Regulations		Date of Data Capture	text(date_mdy)		
4	Country	Regulations		Name of country.	dropdown	*choose from list of countries	
5	reg_any	Regulations		Does this country have any required national **restrictions** that limit exposure or access to unhealthy foods or beverages in the school or surrounding environment (i.e., marketing restrictions, competitive food/beverage standards, school meal standards)?<br><br>**This does NOT include F/V subsidies or incentives, farm-to-school programs, school gardens, or voluntary guidelines.*	yesno	Yes, No	If no, skip to 'comment'
6–25	*These codes were used to review national mandatory* ***restrictions on marketing*** *for unhealthy foods in the school food environment. These results are reported and discussed in a separate manuscript by the same authors.*[17]						
26–57	*These codes were used to review national mandatory* ***restrictions on competitive food sales*** *for unhealthy foods in the school food environment. These results are reported and discussed in a separate manuscript by the same authors.*[17]						

Variable #	Variable/ Field Name	Form Name	Section Header	Field Label	Field Type	Choices, Calculations, OR Slider Labels	Skip Logic
58	meals_prgm	School Meal Regulations	General	Does this country have a national school meals program that is federally or publicly funded?	Checkbox	1, Federally funded national school meals program<br>2, Publicly funded school meal program<br>3, No national school meal program<br>4, Other: Please specify	if 4, go to next question.
59	meals_prgm_other	School Meal Regulations	General	Please specify other:	Text	[open field]	
60	meals_any	School Meal Regulations	General	Does this country have any mandatory national guidelines for meals served to children in schools?	yesno	Yes, No	
61	meals_health	School Meal Regulations	General	Do the national school meal guidelines include criteria that aim to increase the presence, quantity, or consumption of "healthier" or less processed food items?<br><br>**This could include minimum requirement of certain foods groups (i.e. vegetables, protein, etc.), procurement of local F/V, etc.*	yesno	Yes, No	
62	meals_health_notes	School Meal Regulations	General	If so, please provide a brief description of these requirements.	notes	[open field]	show if [meals_health]=1
63	meals_any_restr	School Meal Regulations	General	Do this country's national school meal guidelines include restrictions on types of foods permitted to be served to students through school provided meals?	yesno	Yes, No	If yes, go to next question. If no, skip to END

Variable #	Variable/ Field Name	Form Name	Section Header	Field Label	Field Type	Choices, Calculations, OR Slider Labels	Skip Logic
64	meals_implement	School Meal Regulations	General	Are these regulations currently active?	yesno	Yes, No	
65	meals_implement_notes	School Meal Regulations	General	If not currently active, please provide details.	notes	[open field]	
66	meals_reg_age<br>*meals_reg_age_1<br>*meals_reg_age_2<br>*meals_reg_age_3<br>*meals_reg_age_4	School Meal Regulations	General	To which grades do the school meal restrictions apply?	Checkbox	1, Preschools (pre-kindergarten; under 6 years)<br>2, Primary schools (grades kindergarten-5th grade; ≈6-12 years)<br>3, Middle/Lower Secondary Schools (grades 6-8th; ≈11-13 years)<br>4, High School/Upper Secondary Schools (grades 9-12th or last schooling before university/higher education; ≈13-18 years)	
67	meals_reg_age_notes	School Meal Regulations	General	Include the policy language indicating applicable age/grade range.	text	[open field]	
68	meals_cat	School Meal Regulations	Criteria	Do the school meal restrictions limit specific categories of food (i.e., HFSS, UPFs- ultra processed foods, food groups- potato chips, cakes, sweets, etc.)?	yesno	Yes, No	If yes, go to next question. If no, continue to 'meals_caff_ban'
69	meals_cat_notes	School Meal Regulations	Criteria	If so, please specify categories subject to school meal restrictions.	notes	[open field]	
70	meals_caff	School Meal Regulations	Criteria	Are there regulations on caffeine in beverages served with school meals?	radio	1, Complete ban<br>2, Limits on quantity present in all beverages	

Variable #	Variable/ Field Name	Form Name	Section Header	Field Label	Field Type	Choices, Calculations, OR Slider Labels	Skip Logic
						3, Limits on quantity in specific beverage categories<br>4, No restrictions	
71	meals_caff_spec	School Meal Regulations	Criteria	Please specify specific beverage categories subject to caffeine limits.	notes	[open field]	
72	meals_nns_food	School Meal Regulations	Criteria	Are there school meal regulations on the use of artificial sweeteners/NCS/NNS in foods?	radio	1, Complete ban<br>2, Limits on quantity present in all foods<br>3, Limits on quantity in specific food categories<br>4, No restrictions	If yes, go to next question. If no, continue to 'meals_nns_food'
73	meals_nns_food_spec	School Meal Regulations	Criteria	Please specify specific food categories subject to artificial sweeteners/ NCS/NNS limits.	notes	[open field]	
74	meals_nns_bev	School Meal Regulations	Criteria	Are there school meal regulations on the use of artificial sweeteners/NCS/NNS in beverages?	radio	1, Complete ban<br>2, Limits on quantity present in all foods<br>3, Limits on quantity in specific food categories<br>4, No restrictions	If yes, go to next question. If no, continue to 'meals_nns_bev'
75	meals_nns_bev_spec	School Meal Regulations	Criteria	Please specify specific beverage categories subject to artificial sweeteners/NCS/NNS limits.	notes	[open field]	
76	meals_food_nutr	School Meal Regulations	Criteria	Do the school meals restrictions include nutritional standards for foods?<br><br>*Includes calories, sugar, fat, sodium.	yesno	Yes, No	If yes, go to next question. If no, continue to 'meals_bev_nutr'
77	meals_food_kcal	School Meal Regulations	Criteria	If yes, are there thresholds on total calories?	yesno	Yes, No	

Variable #	Variable/ Field Name	Form Name	Section Header	Field Label	Field Type	Choices, Calculations, OR Slider Labels	Skip Logic
78	meals_food_kcal_category	School Meal Regulations	Criteria	Does the calorie threshold apply:	radio	1, Universally to all foods<br>2, To a specific category|	If 1, skip to 'meals_food_sugar'
79	meals_food_kcal_notes	School Meal Regulations	Criteria	Please specify catgeories:	text	[open field]	
80	meals_food_sugar	School Meal Regulations	Criteria	If yes, are there thresholds on total sugar?	yesno	Yes, No	
81	meals_food_sugar_category	School Meal Regulations	Criteria	Does the sugar threshold apply:	radio	1, Universally to all foods<br>2, To a specific category|	If 1, skip to 'meals_food_addsugar'
82	meals_food_sugar_notes	School Meal Regulations	Criteria	Please specify catgeories:	text	[open field]	
83	meals_food_addsugar	School Meal Regulations	Criteria	If yes, are there thresholds on added sugar?	yesno	Yes, No	
84	meals_food_addsugar_category	School Meal Regulations	Criteria	Does the added sugar threshold apply:	radio	1, Universally to all foods<br>2, To a specific category|	If 1, skip to 'meals_food_totfat'
85	meals_food_addsugar_notes	School Meal Regulations	Criteria	Please specify catgeories:	text	[open field]	
86	meals_food_totfat	School Meal Regulations	Criteria	If yes, are there thresholds on total fat?	yesno	Yes, No	
87	meals_food_totfat_category	School Meal Regulations	Criteria	Does the total fat threshold apply:	radio	1, Universally to all foods<br>2, To a specific category|	If 1, skip to 'meals_food_satfat'
88	meals_food_totfat_notes	School Meal Regulations	Criteria	Please specify catgeories:	text	[open field]	
89	meals_food_satfat	School Meal Regulations	Criteria	If yes, are there thresholds on saturated fat?	yesno	Yes, No	
90	meals_food_satfat_category	School Meal Regulations	Criteria	Does the saturated fat threshold apply:	radio	1, Universally to all foods<br>2, To a specific category|	If 1, skip to 'meals_food_transfat'

Variable #	Variable/ Field Name	Form Name	Section Header	Field Label	Field Type	Choices, Calculations, OR Slider Labels	Skip Logic
91	meals_food_satfat_notes	School Meal Regulations	Criteria	Please specify catgeories:	text	[open field]	
92	meals_food_transfat	School Meal Regulations	Criteria	If yes, are there thresholds on trans fat?	yesno	Yes, No	
93	meals_food_transfat_category	School Meal Regulations	Criteria	Does the sodium threshold apply:	radio	1, Universally to all foods<br>2, To a specific category|	If 1, skip to 'meals_food_sodium'
94	meals_food_transfat_notes	School Meal Regulations	Criteria	Please specify catgeories:	text	[open field]	
95	meals_food_sodium	School Meal Regulations	Criteria	If yes, are there thresholds on sodium?	yesno	Yes, No	
96	meals_food_sodium_category	School Meal Regulations	Criteria	Does the trans fat threshold apply:	radio	1, Universally to all foods<br>2, To a specific category	If 1, skip to 'meals_bev_nutr'
97	meals_food_sodium_notes	School Meal Regulations	Criteria	Please specify catgeories:	text	[open field]	
98	meals_bev_nutr	School Meal Regulations	Criteria	Do the school meals restrictions include nutritional standards for beverages?<br><br>*Includes calories, sugar, fat, sodium.	yesno	Yes, No	If yes, go to next question. If no, continue to 'meals_monitor'
99	meals_bev_thresh<br>*meals_bev_thresh__1<br>*meals_bev_thresh__2<br>*meals_bev_thresh__3<br>*meals_bev_thresh__4<br>*meals_bev_thresh__5<br>*meals_bev_thresh__6<br>*meals_bev_thresh__7	School Meal Regulations	Criteria	Please select all categories with thresholds/nutritional standards for beverages.	Checkbox	1, Total Calories<br>2, Total Sugar<br>3, Added Sugar<br>4, Total Fat<br>5, Saturated Fat<br>6, Trans Fat<br>7, Sodium	
100	meals_monitor	School Meal Regulations	Monitoring & Enforcement	Is there any form of monitoring for these school meal regulations?	yesno	Yes, No	
101	meals_enforce	School Meal Regulations	Monitoring & Enforcement	Is there any form of enforcement of these	yesno	Yes, No	

Variable #	Variable/ Field Name	Form Name	Section Header	Field Label	Field Type	Choices, Calculations, OR Slider Labels	Skip Logic
				marketing regulations?<br><br>Note: "Enforcement" implies actual penalties/punitive action for not abiding by regulations contained with the law or policy. If this is not found, check "no."			
102	meals_me_notes	School Meal Regulations	Monitoring & Enforcement	Please include any relevant information about monitoring or enforcement.	notes	[open field]	
103	comment	General Comments		[NOT REQUIRED] Include any general notes here:<br><br>*This includes: Any ambiguity or uncertainty / missing information; Barriers in the search process; Regional laws that cover most of the country without an official national policy; Whether a country is considering starting or stopping a policy; Any unique policies that are worth highlighting in the paper	notes	[open field]	
104	sources	General Comments		Include hyperlinks for the main sources used to find the information in this form.	notes	[open field]	
105	Zotero	General Comments		Have you entered these sources in Zotero?	radio	1, Yes | 2, Not yet	

References:

1. Food and Agriculture Organization of the United Nations. School food global hub: Fiji. 2022. Accessed August 27, 2024. https://www.fao.org/platforms/school-food/around-the-world/asia-and-the-pacific/fiji/en
2. Food and Agriculture Organization of the United Nations. School food global hub: Finland. Accessed August 27, 2024. https://www.fao.org/platforms/school-food/around-the-world/europe-and-central-asia/finland/en
3. Ministry of Education, Ministry of Health. Health Promoting Schools Policy. Published online 2004. Accessed August 18, 2024. https://www.moe.gov.mv/assets/upload/health_promoting_chool_policy_2004.pdf
4. World Health Organization. Policies in Japan. Global database on the Implementation of Nutrition Action (GINA). Accessed September 27, 2022. https://extranet.who.int/nutrition/gina/en/policies/1461/type_of_policy
5. Ishida H. The History, Current Status, and Future Directions of the School Lunch Program in Japan. *J-Stage*. 2018;76:S2-S11. doi:https://doi.org/10.5264/eiyogakuzashi.76.S2
6. Tanaka N, Miyoshi M. School lunch program for health promotion among children in Japan. *Asia Pac J Clin Nutr*. 2012;21(1):155-158.
7. Morimoto K, Miyahara K. Nutritional Management Implemented at School Lunch Programs in Japan Based on the Changes in Criteria for Provision of School Lunches. *Eiyougakuzashi*. 2018;76(Supplement):S23-S37. doi:10.5264/eiyogakuzashi.76.S23
8. Policy - Syödään ja opitaan yhdessä -kouluruokailusuositus [Eat and Learn Together - School Meal Recommendation]. World Health Organization. Accessed December 22, 2022. https://extranet.who.int/nutrition/gina/en/node/66500
9. Policy actions: Mandatory nutrition standards at state level. World Cancer Research Fund International. Accessed August 18, 2024. https://policydatabase.wcrf.org/level_one?page=nourishing-level-one#step2=1#step3=337
10. World Health Organization. Global database on the Implementation of Food and Nutrition Action (GIFNA). 2024. Accessed August 18, 2024. https://gifna.who.int/
11. World Cancer Research Fund International. NOURISHING and MOVING policy databases. Accessed August 18, 2024. https://policydatabase.wcrf.org/level_one?page=nourishing-level-one
12. Food and Nutrition Service. *National School Lunch Program and School Breakfast Program: Nutrition Standards for All Foods Sold in School as Required by the Healthy, Hunger-Free Kids Act of 2010*.; 2016:50131-50151. Accessed October 18, 2022. https://www.federalregister.gov/documents/2016/07/29/2016-17227/national-school-lunch-program-and-school-breakfast-program-nutrition-standards-for-all-foods-sold-in
13. Food and Nutrition Service, U.S. Department of Agriculture. Nutrition Standards for School Meals. Accessed October 20, 2022. https://www.fns.usda.gov/cn/nutrition-standards-school-meals
14. Food and Nutrition Service. Meal Requirements Under the NSLP & SBP: Q&A for Program Operators Updated to Support the Transitional Standards Effective July 1, 2022. U.S. Department of Agriculture. March 2, 2022. Accessed October 31, 2022. https://www.fns.usda.gov/cn/sp052022-questions-answers-program-operators
15. Kenney EL, Barrett JL, Bleich SN, Ward ZJ, Cradock AL, Gortmaker SL. Impact Of The Healthy, Hunger-Free Kids Act On Obesity Trends. *Health Affairs*. 2020;39(7):1122-1129. doi:10.1377/hlthaff.2020.00133
16. Johnson DB, Podrabsky M, Rocha A, Otten JJ. Effect of the Healthy Hunger-Free Kids Act on the Nutritional Quality of Meals Selected by Students and School Lunch Participation Rates. *JAMA Pediatr*. 2016;170(1):e153918. doi:10.1001/jamapediatrics.2015.3918
17. Perry M, Mardin K, Chamberlin G, et al. National Policies to Limit Food Marketing and Competitive Food Sales in Schools: A Global Scoping Review. *Advances in Nutrition*. 2024;15(8):100254. doi:10.1016/j.advnut.2024.100254
